# Titration Calorimetry Standards and the Precision of Isothermal Titration Calorimetry Data

**DOI:** 10.3390/ijms10062752

**Published:** 2009-06-18

**Authors:** Lina Baranauskienė, Vilma Petrikaitė, Jurgita Matulienė, Daumantas Matulis

**Affiliations:** Laboratory of Biothermodynamics and Drug Design / Institute of Biotechnology, Graičiūno 8, Vilnius, LT-02241, Lithuania; E-Mails: linami@ibt.lt (L.B.); vilma@ibt.lt (V.P.); matuliene@ibt.lt (J.M.)

**Keywords:** titration calorimetry, enthalpy, protein-ligand binding, thermodynamics, carbonic anhydrase

## Abstract

Current Isothermal Titration Calorimetry (ITC) data in the literature have relatively high errors in the measured enthalpies of protein-ligand binding reactions. There is a need for universal validation standards for titration calorimeters. Several inorganic salt co-precipitation and buffer protonation reactions have been suggested as possible enthalpy standards. The performances of several commercial calorimeters, including the VP-ITC, ITC200, and Nano ITC-III, were validated using these suggested standard reactions.

## 1. Introduction

Isothermal titration calorimetry (ITC) provides the most accurate and direct measurement of the enthalpy of any reaction under isothermal and isobaric conditions. It is also the only method capable of determining the enthalpy, entropy, and the Gibbs free energy of a reaction in a single titration experiment. High-sensitivity isothermal titration calorimeters, commercially available since the early 1990s [1], yield an abundance of calorimetric data. Isothermal titration calorimetry has been widely used in fields such as drug discovery to measure the thermodynamic parameters of molecular interactions, including target protein interactions with lead compounds [2–4], protein-DNA interactions [5,6], lipid-DNA interactions [7], lipid-lipid interactions [8], and many others that are well reviewed elsewhere [9–11].

There have been at least two attempts to compile isothermal titration calorimetry data in the form of databases and to correlate thermodynamic data with the structures of bound complexes. The first repository of ITC data was established by Gilson and coworkers [12–14]. The Structure/Calorimetry of Reported Protein Interactions Online database (SCORPIO) of published isothermal titration calorimetry studies and structural information on the interactions between proteins and small-molecule ligands by Ladbury [15] is another nice database where structure-thermodynamics correlations can be studied.

However, the precision, accuracy, and repeatability of some calorimetric data are questionable. Some reported data may not be as accurate or precise as claimed. To determine the precision of ITC data, one can repeat measurements of the same reaction a large number of times in different laboratories. The limitations and the precision of ITC data were demonstrated by comparing the thermodynamic parameters of the binding of several inhibitors to carbonic anhydrase obtained by a number of laboratories worldwide and comparing the ITC results with other methods such as surface plasmon resonance [16,17].

The enthalpy of 4-carboxybenzenesulfonamide (CBS) binding to bovine carbonic anhydrase II was determined by 14 operators of various models of high-sensitivity isothermal titration calorimeters. The resulting value was −10.4±2.5 kcal·mol^–1^ (−43.5±10.5 kJ·mol^−1^). The error of the measurement was surprisingly high and significantly higher than those typically reported for ITC measurements. Despite this large uncertainty, we consider this value to be the most precisely determined enthalpy of a protein-ligand interaction and suggest that any ITC operator studying protein-ligand interactions should check their ITC precision by using this reaction as a standard.

As pointed out by Wadso [18], most ITC users are not particularly concerned about possible systematic errors in their measurements. There have been a number of attempts to determine the scale of the factors that influence accuracy using the statistical error of calorimetric data [19,20], the consistency of calorimetric enthalpies with van’t Hoff enthalpies [21,22], and calorimeter calibration [23,24]. Several associations such as calcium with EDTA, barium with crown ether, ribonuclease A with AMP, and Tris protonation are sometimes used to validate titration calorimeters. However, no reporting of such validation is usually provided.

Here we discuss the need for better and more precisely determined standard reactions to be used for calorimeter validation. Several examples of inaccurate ITC results obtained in our laboratory that appear to be reliable are presented and some possible reasons for the discrepancies are discussed. The accuracy and data precision of several current commercial calorimeters are compared, and the inaccuracies were determined only after careful repetition of the same reactions using several calorimeters.

## 2. Results and Discussion

### 2.1. Enthalpy standards

Isothermal titration calorimeters are calibrated electrically by generating a heat pulse of known power and duration. Such calibration is universal and sufficiently precise. However, the reported data are often inaccurate. Our experience shows that a calorimeter must be validated periodically for precision using the same chemical reaction standards.

The standard reactions can be divided into two groups: reactions involving only chemical reagents and reactions involving ligand binding to proteins. It is important to have a standard reaction of a ligand binding to a protein. However, there are very few reactions where the enthalpy of binding has been determined to high precision and the reagents are readily available from commercial sources. One such reaction is the above-mentioned CBS binding to bovine carbonic anhydrase II. However, despite careful measurements by a number of laboratories worldwide, the uncertainty of the enthalpy is about 25%, and this precision is not sufficient to be used for calorimeter validation.

Another possibility to validate ITC equipment is to use chemical reactions. Several examples are given in Table 1. The advantages of using chemical reactions are that reagents are commercially available in highly purified forms and the enthalpy values are determined with significantly greater precision than protein-ligand interactions. The disadvantages are that chemical reactions used for calorimeter calibration are typically carried out at about 100-fold greater concentrations than the protein-ligand binding reactions of interest and the results may be inaccurate if details such as the presence of CO_2_ in water are unaccounted for. However, with careful preparation of reactants, the reactions listed in Table 1 are good candidates that we propose for calorimeter validation.

**Figure 1.**
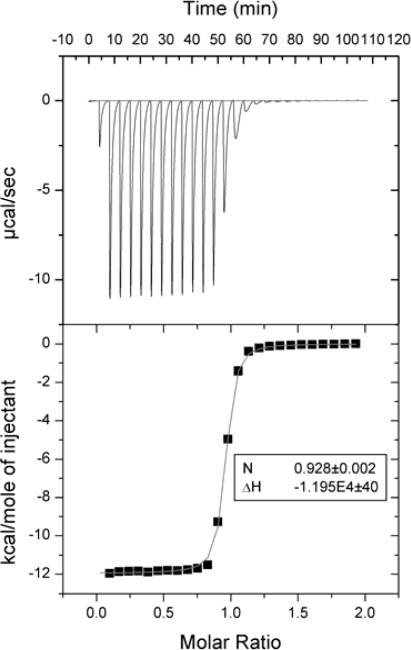
Typical titration of 0.5 mM HNO_3_ with 5 mM Tris base using a VP-ITC (Microcal, Inc.) microcalorimeter at 37 °C. Both the cell and syringe solutions contained 100 mM NaCl.

Figure 1 shows a typical titration of nitric acid with Tris base. The data are nearly perfect, with the stoichiometry and enthalpy within 3% of the literature value. However, even this precision is not sufficient to obtain a reliable heat capacity of binding. Note that this reaction is not suitable for the validation of binding Gibbs free energies determined by ITC. The binding constant is not well determined in this experiment because the factor *c* that must be between 5 and 500 is actually equal to 80,000 (*c* = *nK_b_C*, where *n* – stoichiometry, *K_b_* – binding constant, and *C* – concentration of reactant in the cell, *c* = 1×1.6×10^8^×5×10^−4^ = 8×10^4^). Therefore, the measured binding constant of 4.7×10^5^ M^−1^ is much smaller than the literature value of 1.6×10^8^ M^−1^ (p*K_a_* = 8.2 at 25 °C). However, the validations of binding constants and Gibbs free energies are not the subjects of this manuscript and will not be discussed here.

**Figure 2.**
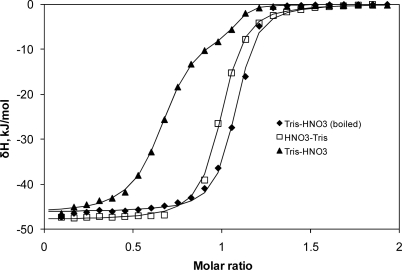
Tris-base – nitric acid ITC titration data obtained with a Microcal VP-ITC calorimeter at 25 °C. Filled symbols: 0.5 mM Tris base in the cell and 5 mM HNO_3_ in the syringe. Open symbols: 0.5 mM HNO_3_ in the cell and 5 mM Tris base in the syringe. All solutions contained 100 mM NaCl. When Tris base is in the cell (0.5 mM), the available concentration is reduced by dissolved CO_2_. Therefore, the stoichiometry was reduced to about 0.7. When Tris base is in the syringe (5 mM), the curve is practically unaffected by CO_2_. Using pre-boiled water in the preparation of Tris base solution solves the problem of reduced stoichiometry. Datapoints are the integrals of ITC raw data and the lines are fitted with Origin 5.0 using one- or two-binding site models.

In order to carry out calorimeter calibration and validation with acid-base titrations as shown in Figure 1 and Table 1, one should carefully degas solutions in order to remove CO_2_. Figure 2 shows Tris acid – base ITC data obtained using conventionally purified water and water that had been pre-boiled to remove dissolved CO_2_. Carbon dioxide reacts with Tris base and reduces the amount that can react with acid. This may be a significant source of error. We emphasize that any reaction where CO_2_ may interfere is not a good standard. This could be one of the reasons why the interaction of CBS with bovine carbonic anhydrase gives such inconsistent results when measurements from various ITC operators are compared. It is possible that the water sources may have contained different concentrations of CO_2_, resulting in competition for binding to the active site of carbonic anhydrase with CBS. However, if the water to be used for solution preparation is pre-boiled, the Tris acid – base reactions are fully suitable for calibration and validation.

**Figure 3.**
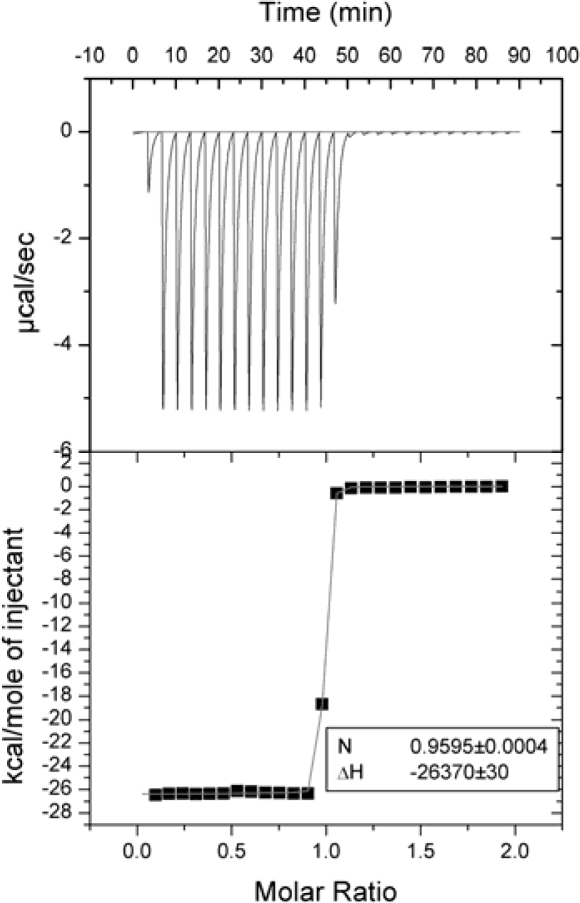
Titration of 0.1 mM NaI with 1.0 mM AgNO_3_ at 25 °C using VP-ITC (Microcal, Inc.) microcalorimeter.

Figure 3 shows typical ITC data from silver halide formation reactions. This reaction is different from protonation reactions that are sometimes used for calorimeter validation. There is no pH change, and the reaction is not dependent on pH near the neutral range used in protein titrations and for many biological molecules. However, sulfhydryl groups and carbonate may interfere. Silver halide formation reaction involves precipitate and thus demonstrates that precipitate formation does not prevent the accurate determination of thermodynamic parameters of binding using ITC. This reaction is a convenient one to use in addition to the Tris – acid titration. We suggest using the reactions listed in Table 1 to validate each calorimeter before reporting measured enthalpies of protein-ligand binding reactions.

### 2.2. Comparison of data obtained using several microcalorimeters

Having carried out over 3,200 titration experiments on a number of isothermal titration calorimeters, including the Omega ITC (Microcal, Inc.), MCS ITC (Microcal, Inc.), VP-ITC (Microcal, Inc.), Auto-ITC (Microcal, Inc.), Nano ITC-III (Calorimetry Sciences Corporation, Inc.), and ITC200 (Microcal Inc.) instruments, we have noticed some systematic inconsistencies among the various models. Commercially available isothermal titration calorimeters have improved their precision and reliability over the years. However, some models are more reliable than others. Microcal calorimeters are somewhat more reliable than those from Calorimetry Sciences Corp., but they are also more expensive. The most recent Microcal ITC200 model is less accurate than the VP-ITC. However, the ITC200 is designed to consume about 5-fold less material than the VP-ITC.

Table 2 lists our results from three calorimeters using the standard reactions listed in Table 1. Standard deviations show the reproducibility of the enthalpy with 95% probability. The values that were most accurate and closest to those reported in the literature were obtained with the VP-ITC, slightly less accurate results were obtained with the ITC200, and the least accurate results were obtained with the Nano ITC-III microcalorimeter.

Interestingly, the Nano ITC-III calorimeter results were very reproducible, but the enthalpy values were systematically underestimated – the reactions should be about 10–20% more exothermic than measured. The silver iodide and silver bromide formation reactions were particularly easy to perform. Silver chloride formation enthalpies and acid-base enthalpies were underestimated both by the Nano ITC-III and the VP-ITC microcalorimeters. Therefore, the most suitable of the studied reactions for calorimeter validation are silver iodide (or bromide) formation and Tris base neutralization with nitric acid.

### 2.3. Uncertainties of protein-ligand binding enthalpies determined by several microcalorimeters

**Figure 4.**
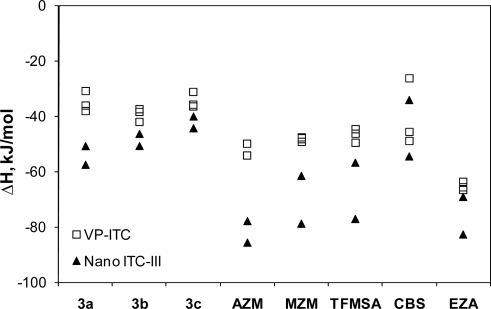
Comparison of measured ligand binding enthalpies to recombinant human carbonic anhydrase II using VP-ITC and Nano ITC-III microcalorimeters. Titrations were performed at 25 °C in 50 mM sodium phosphate buffer, pH 7.0, containing 50 mM NaCl and 1% DMSO. Note that there is a significant systematic overestimation of the enthalpies measured using the Nano ITC-III calorimeter or underestimation of the enthalpies using the VP-ITC calorimeter. All raw titration curves were good with a binding stoichiometry of 0.9±0.1.

Despite careful validation and repetition of the same experiments on a number of calorimeters, the protein-ligand binding data are sometimes quite scattered and do not repeat well. An example of such a situation where two calorimeters give significantly different enthalpy results is shown in Figure 4. Various sulfonamide compounds that are good ligands of human carbonic anhydrase II were titrated using VP-ITC and Nano ITC-III microcalorimeters. There was either a consistent overestimation of the observed binding enthalpy by the Nano ITC-III microcalorimeter (most likely), or underestimation by the VP-ITC microcalorimeter. The most likely explanation for the overestimation by the Nano ITC-III is the instability of the baseline. The manufacturer is currently offering an upgrade for improved baseline stability. It should also be kept in mind that protein-ligand binding reactions were usually carried out with only 1–10 μM protein in the calorimeter cell. These concentrations are much smaller than the reactions described earlier that were used for calorimeter validation and they are near the sensitivity limit of the calorimeters. The Nano ITC-III microcalorimeter appears to yield more reliable results at higher concentrations such as those used for the reactions listed in Table 2. The enthalpies in Table 2 from the Nano ITC-III calorimeter are underestimated by only about 10% compared to literature values, while the enthalpies for reactions at low protein concentration (Figure 4) are overestimated by a larger factor.

Chemical structures of the ligands numbered 3a-3c have been described previously [27]. Other ligand name abbreviations are: AZM – acetazolamide, MZM – methazolamide, TFMSA – trifluoromethane sulfonamide, CBS – p-carboxy benzene sulfonamide, EZA – ethoxzolamide. Calorimetric measurements of their binding to carbonic anhydrases I and II were described previously [28–30].

Such inconsistencies and the large errors of enthalpy determination make it very difficult to discover correlations between compound structure and thermodynamics. The picture is significantly complicated by the linked protonation reactions, as well. After carrying out the linkage analysis [28], the intrinsic binding enthalpies are even less reliable, making it difficult to draw the correct conclusions regarding correlations of structure and thermodynamics.

The main goal of any binding measurement is to gain insights into how the thermodynamic parameters correlate with the structure of bound complex. For example, when we study drug lead compound interactions with a protein target, we are primarily interested in learning which chemical functional groups of the lead compound contribute to the thermodynamic parameters. In other words, we are looking for chemical changes to be made to the lead compound to make the binding more thermodynamically favorable and more specific. Unfortunately, these structure-energy correlations are not well understood and the additivity of various functional group contributions is not straightforward.

There are several reasons for this situation. First, we believe that the precision of ITC data is not sufficient due to poor validation as demonstrated above by comparing results obtained by different calorimeters. Second, many protein-ligand interactions have linked protonation reactions that should be accounted for by performing the binding reactions in several buffers of different protonation enthalpies and at several pH values as explained before [31,32]. Third, determination of the heat capacity by measuring the binding enthalpies at various temperatures should be performed after the protonation linkage analysis at every temperature. These experiments are tedious and time consuming and therefore are often not completely performed.

## 3. Experimental Section

Recombinant human carbonic anhydrase was expressed in *E. coli* and purified as previously described [33]. Carbonic anhydrase ligands were purchased from Sigma-Aldrich Chemical Co., Alfa Aesar, or synthesized as previously described [27]. Chemicals, including salts and buffers (at least 98% chemical purity) were purchased from Sigma-Aldrich Chemical Co.

Isothermal titration calorimetry experiments were carried out with three microcalorimeters: VP-ITC (Microcal, Inc.), ITC200 (Microcal, Inc.), and Nano ITC III (Calorimetry Sciences Corporation, Inc.). Calorimeters were electrically calibrated according to manufacturer’s instructions. Protein or chemical compound (2–500 μM) was loaded into the calorimeter cell as described in the text. The titration syringe was loaded with another reactant at 10- to 20-fold greater concentration than in the cell. Titrations were usually carried out using 20–30 injections of 10 μL each injected at 3–4 minute intervals. Stirring was 150–400 rpm as suggested by the manufacturer. Titrations were carried out at constant temperature in the 13 – 37 ºC temperature range. Both reactant solutions were prepared in a solution containing the same reagents, such as 50 or 100 mM NaCl, as described in the text.

## 4. Conclusions

Isothermal titration calorimetry is the most powerful tool to determine the enthalpies of binding of various reactions, including protonation, coprecipitation, and protein-ligand binding. However, there is a need for more careful validation of all calorimeters, more careful experimental planning regarding the buffers, pHs, and temperatures used, and more careful interpretation of calorimetric data before reporting the thermodynamics of binding of the studied reactions. Our recommendation is to select several reactions, such as 1) Tris base with nitric acid, 2) silver nitrate with sodium iodide or bromide, and 3) bovine carbonic anhydrase II with CBS, and to validate calorimeters by carrying out these reactions. Validation should be reported in the experimental section of every ITC manuscript.

## Figures and Tables

**Table 1.** Chemical reactions that could be used as references for ITC equipment.

**Cell contents**	**Syringe contents**	**Temperature, °C, other conditions**	**Δ*H* (kJ·mol^−1^) from the literature [25,26]**	**Δ*Cp* (J·mol^−1^·K^−1^) from the literature [25,26]**
0.5 mM HNO_3_	5 mM Tris base	25 °C, 100 mM NaCl	−47.45	+73.01
0.5 mM HNO_3_	5 mM NaOH	25 °C, 100 mM NaCl	−55.81	+223.85
0.2 mM NaCl	2 mM AgNO_3_	25 °C	−65.72	+165.39
0.2 mM NaBr	2 mM AgNO_3_	25 °C	−84.75	+172.38
0.2 mM NaI	2 mM A1gNO_3_	25 °C	−110.9	+177.32

**Table 2.** Comparison of results obtained with standard reactions listed in Table 1 using various isothermal titration calorimeters.

**Reaction**	**Literature values (Table 1), kJ·mol^−1^**	**Δ*H*, kJ·mol^−1^, obtained in our laboratory withthese calorimeters**		
		**VP-ITC**	**ITC200**	**Nano ITC-III**
Tris-base + HNO_3_ → Tris-acidic + …, 25 °C	−47.45	−47.8 ± 1.0	−48.4 ± 2.1	−41.9 ± 0.6
Tris-base + HNO_3_ → Tris-acidic + …, 13 °C	−48.33	−47.5 ± 0.8	−47.2 ± 3.4	−44.4 ± 0.1
Tris-base + HNO_3_ → Tris-acidic + …, 37 °C	−46.57	−46.9 ± 0.9	−50.0 ± 2.8	−40.8 ± 0.1
NaOH + HNO_3_ → H_2_O + …, 25 °C	−55.81	−52.6 ± 1.8	ND	−47.2 ± 2.3
AgNO_3_ + NaCl → AgCl↓ + …, 25 °C	−65.72	−57.9 ± 2.0	ND	−52.3 ± 2.6
AgNO_3_ + NaBr → AgBr↓ + …, 25 °C	−84.75	−84.4 ± 3.1	ND	ND
AgNO_3_ + NaI → AgI↓ + …, 25 °C	−110.9	−110.0 ± 1.7	−103.4 ± 10.8	ND

ND – not determined. At least three repeats were carried out and, in most cases, at a number of concentrations to determine the best conditions to most closely match values in Table 1.
